# Systematic Variation in the Pattern of Gene Paralog Retention between the Teleost Superorders Ostariophysi and Acanthopterygii

**DOI:** 10.1093/gbe/evu074

**Published:** 2014-04-14

**Authors:** Daniel Garcia de la serrana, Edson A. Mareco, Ian A. Johnston

**Affiliations:** ^1^School of Biology, Scottish Oceans Institute, University of St Andrews, Fife, United Kingdom; ^2^Department of Morphology, Institute of Biosciences of Botucatu, UNESP—Univ Estadual Paulista, São Paulo, Brazil

**Keywords:** fish evolution, ploidy, gene loss, whole-genome duplication

## Abstract

Teleost fish underwent whole-genome duplication around 450 Ma followed by diploidization and loss of 80–85% of the duplicated genes. To identify a deep signature of this teleost-specific whole-genome duplication (TSGD), we searched for duplicated genes that were systematically and uniquely retained in one or other of the superorders Ostariophysi and Acanthopterygii. TSGD paralogs comprised 17–21% of total gene content. Some 2.6% (510) of TSGD paralogs were present as pairs in the Ostariophysi genomes of *Danio rerio* (Cypriniformes) and *Astyanax mexicanus* (Characiformes) but not in species from four orders of Acanthopterygii (Gasterosteiformes, *Gasterosteus aculeatus*; Tetraodontiformes, *Tetraodon nigroviridis*; Perciformes, *Oreochromis niloticus*; and Beloniformes, *Oryzias latipes*) where a single copy was identified. Similarly, 1.3% (418) of total gene number represented cases where TSGD paralogs pairs were systematically retained in the Acanthopterygian but conserved as a single copy in Ostariophysi genomes. We confirmed the generality of these results by phylogenetic and synteny analysis of 40 randomly selected linage-specific paralogs (LSPs) from each superorder and completed with the transcriptomes of three additional Ostariophysi species (*Ictalurus punctatus* [Siluriformes], *Sinocyclocheilus* species [Cypriniformes], and *Piaractus mesopotamicus* [Characiformes]). No chromosome bias was detected in TSGD paralog retention. Gene ontology (GO) analysis revealed significant enrichment of GO terms relative to the human GO SLIM database for “growth,” “Cell differentiation,” and “Embryo development” in Ostariophysi and for “Transport,” “Signal Transduction,” and “Vesicle mediated transport” in Acanthopterygii. The observed patterns of paralog retention are consistent with different diploidization outcomes having contributed to the evolution/diversification of each superorder.

## Introduction

Polyploidy, involving whole-genome duplication (WGD) and the doubling of gene content, is considered a major feature of the evolution of eukaryotic genomes (Taylor et al. 2003). WGD is usually followed by diploidization and the loss of gene paralogs, a process that may occur over a protracted period (Brunet et al. 2006; Kasahara et al. 2007). Signatures of ancient polyploidy events are evident in many eukaryotic genomes (Jiao et al. 2011; Zhan et al. 2014). For example, the ancestral genome of vertebrates is thought to have undergone two consecutive rounds (1R/2R) of WGD (Dehal and Boore 2005), with a third round (3R) in the lineage leading to teleost fish (Taylor et al. 2003; Jaillon et al. 2004). WGD at the base of the teleost fish radiation (teleost-specific whole-genome duplication [TSGD]) was estimated at 450–320 Ma (3R) (Kuraku and Meyer 2009; Sato and Nishida 2010). It is thought that around 15% of TSGD paralogs have been retained in the diploid genome of modern species (Braasch and Postlethwaite 2012). Several mechanisms have been suggested to explain the retention of paralogs after WGD or small-scale duplications including the appearance of mutations leading to altered regulation (subfunctionalization) and/or the evolution of some novel function (neofunctionalization), which confers a selective advantage (Maere and Van de Peer 2010). Thus, polyploidy contributes to an increase in gene content and at some level has likely contributed to the evolutionary success of modern day taxa. For example, it has been argued that WGD promotes speciation via divergent resolution where the loss of different copies of duplicated genes in allopatric populations leads to genetic isolation (Taylor et al. 2001).

However, the extent to which specific polyploidy events contribute to evolutionary success and speciation is a matter of long-standing debate. For example, it has been estimated that approximately 88% of teleost species are of recent origin, such that the TSGD event may explain as little as 10% of the total diversity (Santini et al. 2009). Similarly, a well-constrained estimate of the salmonid WGD (4R) placed it at 88 Ma, whereas the subfamilies emerged 40–50 Ma and 50% of species formed within the last 5 Ma (Macqueen and Johnston 2014). These results at least indicate a major decoupling between WGD and species diversification while not excluding long-lasting effects of the ploidy event.

Present day examples of polyploidy are particularly widespread in the plant kingdom (Bowers et al. 2003; Jiao et al. 2011). Polyploid lineages are also relatively common in teleosts (Zhan et al. 2014) and have been reported in some amphibians and reptiles (Mable et al. 2011). Phylogenetic studies have shown that recently formed plant lineages experience lower diversification rates relative to diploid congeners as a consequence of both lower speciation and extinction rates (Zhan et al. 2014). In contrast, using comparable methods in teleost, similar diversification rates have been found between polyploid and diploid relatives in some cases (Acipenseridae, Botiidae [families], Salmoniformes [order]), whereas the subfamily Cyprininae revealed higher polyploid diversification [Zhan et al. 2014]).

This study aimed to exploit the recent increase in teleost genome and large-scale transcriptomic data sets to provide an insight into the role of ancient polyploidy on subsequent diversification of teleosts. We tested the hypothesis that different diploidization outcomes have occurred between two of the main teleost superorders: The Ostariophysi and Acanthopterygii by searching for a systematic difference in the retention of TSGD gene paralogs. We further investigated whether there were either chromosomal or functional biases in the retained paralogs between lineages.

## Results and Discussion

A previous comprehensive phylogenetic analysis using 42 orthologous nuclear protein-coding genes estimated that the split of the Euteleostei superorders the Ostariophysi and Acanthopterygii took place in the early Triassic 217 Ma (Steinke et al. 2006). In this study, a comparison of Acanthopterygii and Ostariophysi proteomes (see Materials and Methods and supplementary fig. S1, Supplementary Material online) revealed that 21% of the total genes analyzed (4,122 out of 19,600) in the Ostariophysi superorders and 18.4% (3,284 out of 17,800) in the Acanthopterygii superorders were present as TSGD paralogs, in agreement with previous estimates (Braasch and Postlethwaite 2012).

**Fig. 1.—:**
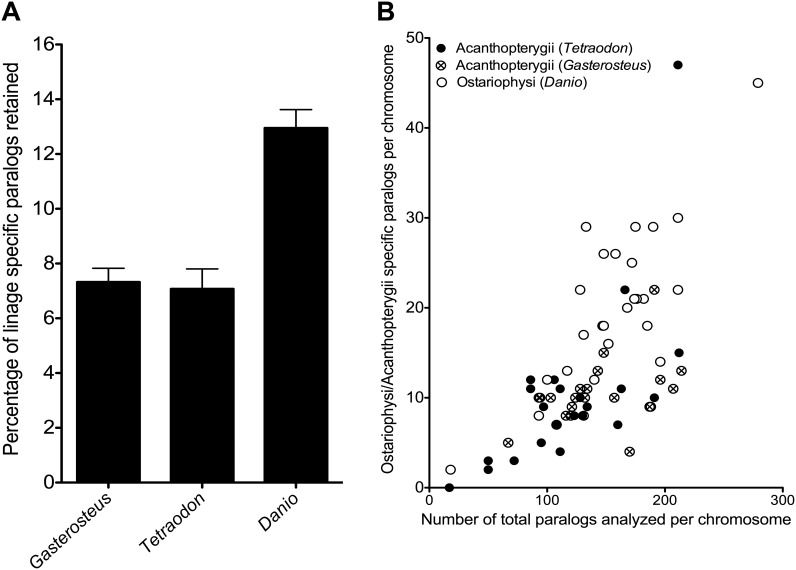
Ostariophysi- and Acanthopterygii-LSP retention and chromosome distribution. (*A*) Percentage of LSP retained over the total of TSGD paralogs analyzed in *Gasterosteus aculeatus* (*n* = 21 chromosomes), *Tetraodon nigroviridis* (*n* = 21 chromosomes), and *Danio rerio* (*n* = 25 chromosomes). Values represent average of chromosomes LSPs ± standard error. (*B*) Correlation plot between number of TSGD paralogs in each *D. rerio* (empty circles), *T. nigroviridis* (filled circles), and *G. aculeatus* (crossed circles) chromosome against the number of LSP identified in the same chromosome; Spearman correlation (ρ) and statistical significance are shown.

**Fig. 2.—:**
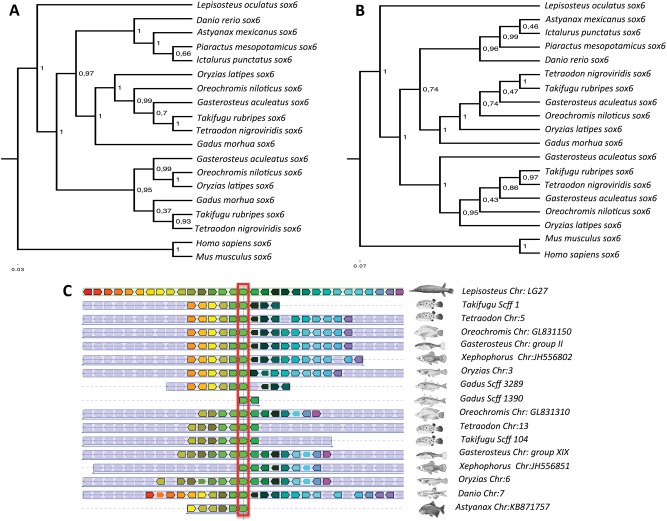
Phylogenetic (*A*, *B*) and synteny (*C*) analysis for LSP from Acanthopterygii species. (*A*) Bayesian phylogenetic relationships for the *Sex Determination Region Y box 6* gene (*sox6*). Tree nodes values represent posterior values. (*B*) Maximum likelihood phylogenetic relationships for *sox6.* Phylogenetic trees nodes values represent posterior values. (*C*) Synteny of the Acanthopterygii LSP of *sox6* across teleost species*.* Genes are indicated as colored boxes, and orthologs share the same color. To aid interpretation, all *sox6* orthologs were aligned and are highlighted in red.

**Fig. 3.—:**
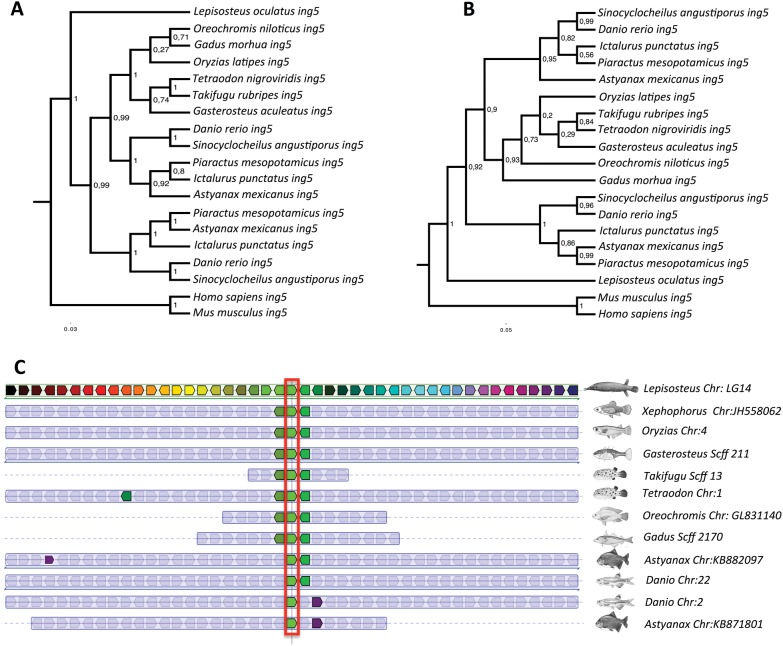
Phylogenetic (*A*, *B*) and synteny (*C*) analysis for LSP from Ostariophysi species. (*A*) Phylogenetic relationships for the *inhibitor of growth protein 5* (*ing5*) gene. Tree node values represent posterior values. (*B*) Maximum likelihood phylogenetic relationships for *ing5.* Phylogenetic trees nodes values represent posterior values. (*C*) Synteny of the Ostariophysi LSP of *ing5* across teleost species*.* Genes are indicated as colored boxes, and orthologs share the same color. To aid interpretation, *ing5* orthologs were aligned and are highlight in red.

The superorder Ostariophysi comprises five orders (Gonorynchiformes, Cypriniformes, Characiformes, Siluriformes, and Gymnotiformes) containing 6,507 species (Nelson 2006). The Ostariophysi are characterized by the Weberian apparatus consisting of modified vertebrae, which connect and transmit sound waves from the swim bladder to the inner ear to increase hearing sensitivity. Genome sequences are only currently available for the zebrafish (*Danio rerio*) and the blind cave fish (*Astyanax mexicanus*) belonging to the orders Cypriniformes and Characiformes, respectively (Steinke et al. 2006). Gene orthologs were identified that occurred as paralogous pairs on different chromosomes in Ostariophysi but were present as singletons in four species of Acanthopterygii from different orders (Gasterosteiformes, *Gasterosteus aculeatus*; Tetraodontiformes, *Tetraodon nigroviridis*; Perciformes, *Oreochromis niloticus*; and Beloniformes, *Oryzias latipes*). This yielded a list of 205 candidate orthologs present as 510 TSGD paralogs (2.6% of total genes analyzed) in Ostariophysi but not Acanthopterygii species (fig. 1*A*; supplementary file S2, Supplementary Material online). Those cases in which both paralogs were retained in one linage but a single copy in the other were considered potential linage-specific paralogs (LSPs). To further investigate the possibility that these orthologous have been retained as paralogous throughout the suborder, we carried out phylogenetic and synteny analysis on a subset of 40 randomly selected Ostariophysi LSPs. The phylogenetic analysis was completed with orthologs from three further Ostariophysi species the catfish *Ictalurus punctatus* (order Siluriformes), *Sinocyclocheilus* species (Cypriniformes) retrieved from the National Center for Biotechnology Information (NCBI) transcriptome database, (www.ncbi.nlm.nih.gov, last accessed March 15, 2014, Transcriptomic Shotgun Assembly), and the pacu *Piaractus mesopotamicus* (Characiformes) (Mareco EA et al., unpublished data) (supplementary files S3 and S4, Supplementary Material online). Although only three of the five Ostariophysi orders were examined, all the 40 selected orthologous were present as paralog pairs in basal and more derived species, consistent with their retention throughout the superorder (fig. 2). Using a similar rational, we found that orthologs of 113 genes representing 226 TSGD paralogs (1.3% of total gene content) were systematically retained in all Acanthopterygii genomes tested (*Oreochromis, Tetraodon, Oryzias,* and *Gasterosteus*) but as a single copy in the two Ostariophysi genomes available (*Danio* and *Astyanax*). The Acanthopterygii sampled included Perciformes and Beloniformes, which split 113 Ma and last shared a common ancestor with the Tetraodontiformes 195 Ma (Steinke et al. 2006). Nevertheless, the result for the Acanthopterygii superorder is less robust than for the Ostariophysi because we only sampled 4 of the 13 orders existent (Nelson 2006). Similarly, phylogenetic and synteny analysis was carried out in a subset of 40 random Acanthopterygii LSPs (fig. 3). LSPs identified in *Tetraodon*, *Gasterosteus,* and *Danio* chromosomes were proportional to the number of TSGD-paralogs analyzed per chromosome (fig. 1*B*; ρ = 0.632; *P* = 0). This result indicates that the putative superorder-specific paralogs were not retained on specific chromosomes or had originated from chromosome-specific rearrangements.

To gain an insight into any potential functional consequences of these difference paralog retention patterns, we performed a gene ontology (GO) SLIM enrichment analysis for the Ostariophysi and Acanthopterygii LSPs relative to the human GO SLIM database. A significant enrichment in GO terms related to “Development,” “Growth,” and “Cell differentiation” was found in the Ostariophysi, whereas the Acanthopterygii showed significant differences in “Signal transduction,” “Transport,” and the “Vesicle mediated transport” (table 1 and supplementary file S5, Supplementary Material online).

**Table 1:** GO Enrichment Analysis of Ostariophysi and Acanthopterygii LSPs

	GO SLIM Term Enriched	GO ID	*P*-value	Number of Genes
Ostariophysi LSPs				
Biological process	Embryo development	0009790	2.7E^−4^	24
	Growth	0040007	1.7E^−2^	10
	Anatomical structure development	0048856	2.3E^−2^	55
	Cell differentiation	0030154	3.0E^−2^	40
Molecular function	DNA binding	0003677	8.4E^−3^	42
	Nucleic acid binding transcription factor	0001071	1.0E^−2^	21
Cell component	Nucleus	0005634	6.13E^−3^	87
	Cytosol	0005829	2.3E^−2^	40
Acanthopterygii LSPs				
Biological process	Signal transduction	0007165	3.3E^−3^	32
	Vesicle-mediated transport	0016192	8.2E^−3^	11
	Anatomical structure development	0048856	1.3E^−2^	29
	Response to stress	0006950	1.5E^−2^	24
	Transport	0006810	4.9E^−2^	22
Cell component	Cytoplasm	0005737	7.6E^−4^	59
	Golgi apparatus	0005794	2.1E^−3^	14

Note.—GO ID, gene ontology identifier. Only GO levels with more than ten genes are shown.

A further inspection of the gene lists in table 1 allowed us to identify some of the individual genes within the GO terms that were significantly different for each superorder. Ostariophysi species have retained two copies of key transcription factors involved in development including members of the Hox gene family (*hoxc6, hoxc11, hoxc12,* and *hoxc13*) involved in patterning (Mallo et al. 2010), Sox gene family members (*sox1*, *sox19* and *sox21*) with diverse developmental functions (Sarkar and Hochedlinger 2013), and six family members (*six1, six2* and *six4*) involved in DNA-binding specificity and in mediating protein–protein interactions (Kumar 2009). In all these cases, only a single TSGD paralog was retained in the Acanthopterygii genomes analyzed. Similarly, Ostariophysi have retained duplicated genes from the PI3K/IGF/mTOR pathway (*rictor, rps6ka3, igf2,* and *igf2bp2*), which is involved in growth and protein synthesis (reviewed in Johnston et al. 2011). In contrast, Acanthopterygii have retained two copies of some Rab GTPases (*rab9a*, *rab19*, *rab27b*, *rab5c,* and *rab8a*), which have a role in membrane trafficking including vesicle formation and movement and membrane fusion (McCormick 1995).

In summary, we provide evidence for systematic differences in TSGD paralog retention between the teleost superorders Ostariophysi and Acanthopterygii of the order of 1–2% of gene content. The scale of these differences and preliminary GO analysis indicate a persistent signature of the TSGD event that may be of functional significant for the subsequent evolution/diversification of each lineage. Our results are consistent with a lingering influence of the TSGD on speciation. Continuous advances in sequencing technology will increase the number and diversity of genomes available enabling further testing of the hypothesis of large-scale conservation of paralog retention between different branches of the ray-finned teleost radiation.

## Conclusions

This study has shown that some TSGD paralogs have been systematically retained in Acanthopterygii but Ostariophysi superorders of teleosts (1.3% and 2.6% of total gene content, respectively). We also showed that LSPs are randomly distributed in teleost genomes, but there were significant differences in the retention of key genes related to growth and embryonic development between the superorders, which may have influenced their subsequent evolution.

## Materials and Methods

### Identification of LSPs

The method for identifying TSGD paralogs that have been systematically retained as pairs in one superorder but as a single copy in the other is schematically illustrated in supplementary figure S1, Supplementary Material online. Among fish with sequenced genomes, *D. rerio* and *G. aculeatus* are the Acanthopterygii and Ostariophysi species, respectively, that have the highest numbers of annotated gene sequences. The first step in our analysis involved reciprocal BLASTs of the proteomes from *D. rerio* (www.ensembl.org, last accessed March 15, 2014; vZv9) and *G. aculeatus* (www.ensembl.org, last accessed March 15, 2014; v.BROADS1) using the BLASTp algorithm included in BioEdit software (http://www.mbio.ncsu.edu/bioedit/bioedit.html, last accessed April 22, 2014) with an *e*-value cutoff of E^−^^80^. A total of 19,600 and 17,800 positive hits were obtained from Ostariophysi/Acanthopterygii and Acanthopterygii/Ostariophysi comparisons, respectively. Those genes annotated as TSGD paralogs, and their ortholog from each of the comparisons, were manually retrieved from both lists based on their Ensembl annotation (www.ensembl.org, last accessed March 15, 2014). Putative TSGD paralogs were aligned using ClustalW to verify that chimeras, splice variants, or isoforms were excluded from the analysis. The remaining duplicated sequences that met the twin criteria of occurring on different chromosomes and existing as a single ortholog in *Lepisosteus oculatus* genome (a pre-TSGD teleost; www.ensembl.org, last accessed March 15, 2014; vLepocu1) and in human (*Homo sapiens*) genome (www.ensembl.org, last accessed March 15, 2014; vGRCh37.p13) were considered to be genuine TSGD paralogs.

To identify those duplicates that were systematically retained in Ostariophysi superorder, *D. rerio* paralogs with a single best hit against the same *G. aculeatus* (Gasterosteiformes) ortholog were retrieved. To identify genes present as duplicates in other Ostariophysi and singletons in Acanthopterygii, the *D. rerio* gene list was blasted against the *A. mexicanus* (vAstmex102) (www.ensembl.org, last accessed March 15, 2014) (Characiformes) and three more Acanthopterygii genomes (*O. latipes* [Beloniformes; v.HdrR, www.ensembl.org, last accessed March 15, 2014], *T. nigroviridis* [Tetraodontiformes; v.TETRAODON8.0, www.ensembl.org, last accessed March 15, 2014], *O. niloticus* [Perciformes; v.Orenil1.0, www.ensembl.org, last accessed March 15, 2014]). Those TSGD with two orthologs in *Astyanax* and *Danio,* but a single copy in all four Acanthopterygii genomes, were considered as Ostariophysi LSPs. Phylogenetic and synteny analysis was carried out using 40 randomly selected LSPs from the Ostariophysi superorder. Because only two Ostariophysi genomes are available, transcriptomic data from representative species from three other Ostariophysi species were used to increase the power of the analysis (*I. punctatus* [Siluriformes] [www.ncbi.nlm.nih.gov, last accessed March 15, 2014], *Sinocyclocheilus* [Cypriniformes] [www.ncbi.nlm.nih.gov, last accessed March 15, 2014], and *P. mesopotamicus* [Characiformes] [Mareco EA et al., unpublished data]). In some cases, it was not possible to include data of all three Ostariophysi-species due to limitations in the transcriptomic database. Transcriptomes are based on expressed genes present in an organism in a specific physiological stage, which means that lowly expressed genes are often missed (as an example see Garcia de la Serrana et al. 2012). However, for all phylogenetic trees generated, there was at least one species present from each of the three Ostariophysi orders. All the amino acid sequences used for phylogenetic analysis are provided in supplementary file S6, Supplementary Material online. To identify those genes that occurred as duplicates in Acanthopterygii but singletons in Ostariophysi, we filtered the TSGD paralogs from *G. aculeatus* against successive rounds of BLAST against the genomes of Acanthopterygians *T. nigroviridis, Ory. latipes*, *O. niloticus,* and the Ostariophysi *A. mexicanus*. Those TSGD paralogs that were present as duplicates in species from all four orders but single copy in *Astyanax* and *Danio* were considered as Acanthopterygii LSPs. Similarly, a subset of 40 randomly selected LSPs were used for phylogenetic and synteny analysis. Similarly, phylogenetic analysis was completed with transcriptomic data from *Ictalurus, Piaractus,* and *Sinocyclocheilus.*

### Phylogenetic Analysis

Peptides sequences were aligned using the GUIDANCE online server (Penn et al. 2010) with PRANK as multisequence alignment algorithm. Columns below the 0.93 Guidance score cutoff were removed from the final alignment used for the phylogenetic trees construction (all alignments are provided in supplementary file S7, Supplementary Material online). Bayesian MCMC phylogenetic trees, following a Yule speciation process model and UPGMA starting tree, were generated for each alignment using BEAST v1.7.5 software with 5,000,000 random seeds (Drummond et al. 2012). Guidance alignments were also used to construct maximum likelihood (ML) phylogenetic trees for each of the LSP analyzed. ML trees were constructed using PhyML online server (http://www.atgc-montpellier.fr/phyml/, last accessed March 12, 2014) (Dereeper et al. 2008). The best evolutionary model for each alignment used to calculate the phylogenetic trees was determined by MEGA5 software (Tamura et al. 2010). Final Bayesian trees were generated using TreeAnnotator v1.7.5 with a burnin value of 1,000. All trees were visualized using FigTree v1.3.1.

### Synteny and GO Analysis

Synteny surrounding Ostariophysi and Acanthopterygii LSPs used for the phylogenetic analysis were inferred using the Genomicus webserver (www.genomicus.biologie.ens.fr) (Louis et al. 2013). For the GO analysis, each list of LSPs was individually analyzed against the human GO database (with the most extensive annotation) and for enrichment analysis, using the STRING sever (www.string-db.org, last accessed March 8, 2014) (Franceschini et al. 2013). To give a broad overview of the ontology content without the details of the specific fine-grained GO terms, the GO Slim annotation was used to classify enriched GO terms.

### Statistical Analysis

Because the distribution of TSGD and LSP paralogs was homogenous, we use the ratio for each chromosome as a pseudoreplicate to calculate the average and standard deviation of LSP retention in Ostariophysi and Acanthopterygii lineages. Spearman correlation between TSGD paralogs and LSPs per chromosome was calculated using SPSS21 statistics package (IBM).

## Supplementary Material

Supplementary files S1–S7 are available at *Genome Biology and Evolution* online (http://www.gbe.oxfordjournals.org/).

Supplementary Data
