# Supplementary material for: Systematic Variation in the Pattern of Gene Paralog Retention between the Teleost Superorders Ostariophysi and Acanthopterygii
Source: Genome Biol Evol. 2014 Apr 14;6(4):981–7. doi: 10.1093/gbe/evu074 (PMC4007551; doi:10.1093/gbe/evu074)
Supplement: Supplementary Data [file supp_6_4_981__index.html]

Systematic Variation in the Pattern of Gene Paralog Retention between the Teleost Superorders Ostariophysi and Acanthopterygii — Supplementary Data 

# Systematic Variation in the Pattern of Gene Paralog Retention between the Teleost Superorders Ostariophysi and Acanthopterygii

## Supplementary Data

files

**Files in this Data Supplement:**

- Supplementary Data - zip file
