# Supplementary material for: Systematic Variation in the Pattern of Gene Paralog Retention between the Teleost Superorders Ostariophysi and Acanthopterygii
Source: Genome Biol Evol. 2014 Apr 14;6(4):981–7. doi: 10.1093/gbe/evu074 (PMC4007551; doi:10.1093/gbe/evu074)
Supplement: Supplementary Data [file supp_evu074_suppl_data.zip › Supplementary_figure1.pptx]

## Slide 1
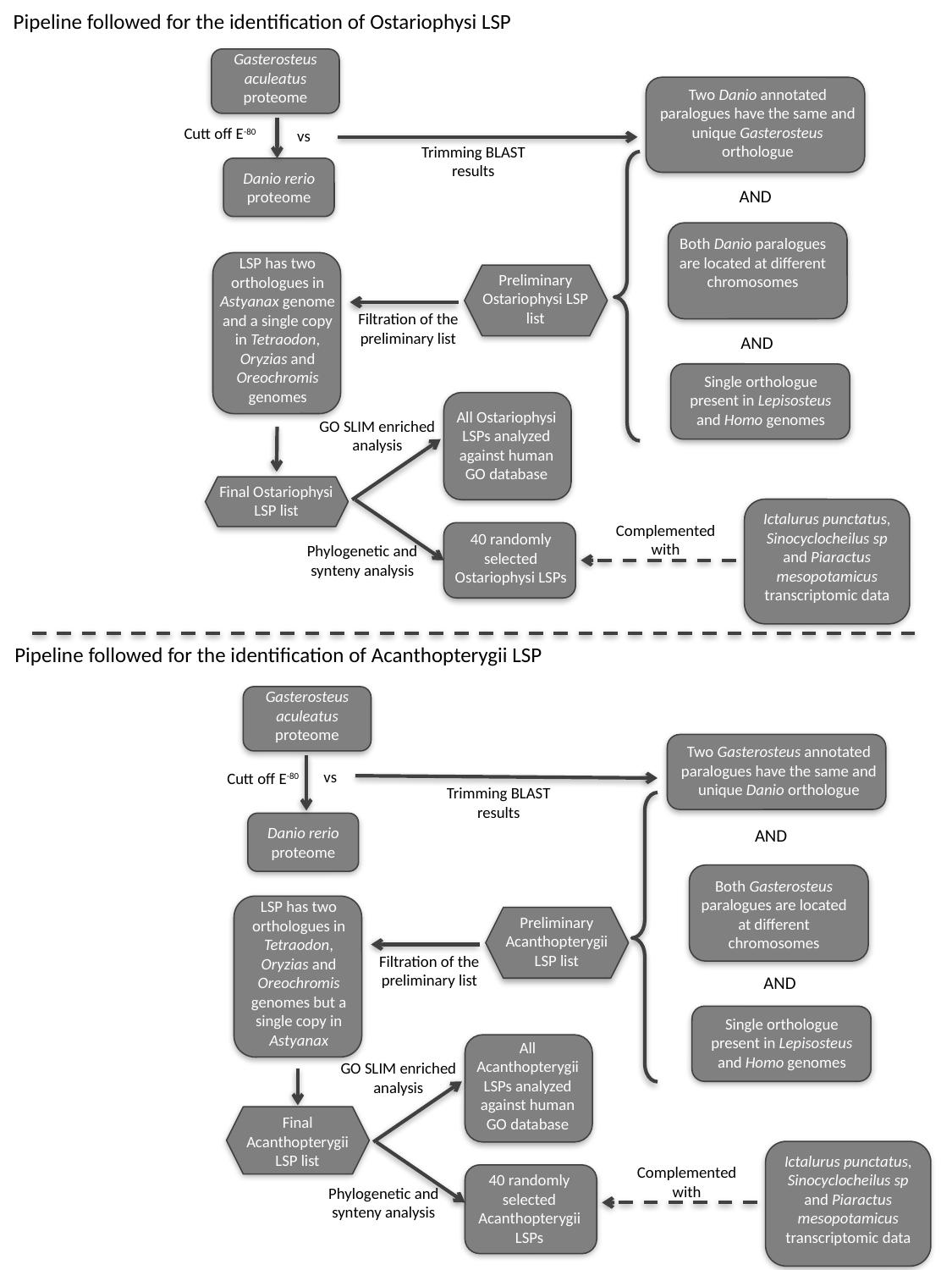

Pipeline followed for the identification of Ostariophysi LSP
Gasterosteus aculeatus proteome
Two Danio annotated paralogues have the same and unique Gasterosteus orthologue
Cutt off E-80
vs
Trimming BLAST results
Danio rerio proteome
AND
Both Danio paralogues are located at different chromosomes
LSP has two orthologues in Astyanax genome and a single copy in Tetraodon, Oryzias and Oreochromis genomes
Preliminary Ostariophysi LSP list
Filtration of the preliminary list
AND
Single orthologue present in Lepisosteus and Homo genomes
All Ostariophysi LSPs analyzed against human GO database
GO SLIM enriched analysis
Final Ostariophysi LSP list
Ictalurus punctatus, Sinocyclocheilus sp and Piaractus mesopotamicus transcriptomic data
Complemented with
40 randomly selected Ostariophysi LSPs
Phylogenetic and synteny analysis
Pipeline followed for the identification of Acanthopterygii LSP
Gasterosteus aculeatus proteome
Two Gasterosteus annotated paralogues have the same and unique Danio orthologue
vs
Cutt off E-80
Trimming BLAST results
Danio rerio proteome
AND
Both Gasterosteus paralogues are located at different chromosomes
LSP has two orthologues in Tetraodon, Oryzias and Oreochromis genomes but a single copy in Astyanax
Preliminary Acanthopterygii LSP list
Filtration of the preliminary list
AND
Single orthologue present in Lepisosteus and Homo genomes
All Acanthopterygii LSPs analyzed against human GO database
GO SLIM enriched analysis
Final Acanthopterygii LSP list
Ictalurus punctatus, Sinocyclocheilus sp and Piaractus mesopotamicus transcriptomic data
Complemented with
40 randomly selected Acanthopterygii LSPs
Phylogenetic and synteny analysis
